# Transcriptomic Analysis Suggests the M1 Polarization and Launch of Diverse Programmed Cell Death Pathways in Japanese Encephalitis Virus-Infected Macrophages

**DOI:** 10.3390/v12030356

**Published:** 2020-03-24

**Authors:** Zhao-Yang Wang, Zi-Da Zhen, Dong-Ying Fan, Pei-Gang Wang, Jing An

**Affiliations:** 1 Department of Microbiology, School of Basic Medical Sciences, Capital Medical University, Beijing 100069, China; 18611000168@163.com (Z.-Y.W.); 15801574235@163.com (Z.-D.Z.); dengue@ccmu.edu.cn (D.-Y.F.); 2 Center of Epilepsy, Beijing Institute for Brain Disorders, Beijing 100093, China

**Keywords:** Japanese encephalitis virus, transcriptomic analysis, macrophage polarization, programmed cell death, oxidative stress

## Abstract

The Japanese encephalitis virus (JEV) is a *Culex* mosquito-borne flavivirus and is the pathogenic agent of Japanese encephalitis, which is the most important type of viral encephalitis in the world. Macrophages are a type of pivotal innate immunocyte that serve as sentinels and respond quickly to pathogen invasions. However, some viruses like JEV can hijack macrophages as a refuge for viral replication and immune escape. Despite their crucial involvement in early JEV infection, the transcriptomic landscapes of JEV-infected macrophages are void. Here, by using an in situ JEV infection model, we investigate the transcriptomic alteration of JEV-infected peritoneal macrophages. We found that, upon JEV infection, the macrophages underwent M1 polarization and showed the drastic activation of innate immune and inflammatory pathways. Interestingly, almost all the programmed cell death (PCD) pathways were activated, especially the apoptosis, pyroptosis, and necroptosis pathways, which were verified by the immunofluorescent staining of specific markers. Further transcriptomic analysis and TUNEL staining revealed that JEV infection caused apparent DNA damage. The transcriptomic analysis also revealed that JEV infection promoted ROS and RNS generation and caused oxidative stress, which activated multiple cell death pathways. Our work uncovers the pivotal pathogenic roles of oxidative stress and multiple PCD pathways in JEV infection, providing a novel perspective on JEV–host interactions.

## 1. Introduction

Japanese encephalitis virus (JEV) taxonomically belongs to the family *Flaviviridae* and the genus *Flavivirus*. Compared with other flaviviruses, such as dengue virus (DENV) and Zika virus (ZIKV), JEV is much more neurotropic [1]. The infection of JEV in the brain causes severe encephalitis, named Japanese encephalitis (JE). JE is the top type of viral encephalitis in the world, posing a threat to the health of more than two billion people in twenty-four countries. The estimated annual case number is 69,000, with a global incidence of 1.8/100,000, causing 10,000–15,000 mortalities [2]. The case fatality of JE is 20% and can be 30% in children. Even after surviving encephalitis, about 50% of patients develop permanent neuropsychiatric sequelae. The disease burden of JE has exceeded dengue and ranks first among arboviral encephalitis in the world [1]. Despite its perniciousness, we have very limited knowledge about the pathogenesis and specific therapeutics of JE.

Macrophages are a type of innate immunocyte that play crucial roles in the immune and inflammatory responses to pathogen infections [3]. Despite their strong abilities in pathogen killing and antigen presentation, macrophages can also be hijacked by a range of pathogens as a refuge for propagation and immune escape [4]. JEV is a pathogen that hijacks macrophages for early replication, immune escape, and pathogenesis. Macrophages at the sites of mosquito bites are the first target cells for JEV, supporting viral amplification and spreading to lymph nodes where the large-scale replication of viruses takes place. Macrophages express IgG Fc receptors, which may enhance the endocytosis of IgG bound viruses and promote viral replication and spread [5]. Upon infection or other stimulation, macrophages undergo a series of molecular and functional differentiations, which are termed polarization. Classical M1 and alternative M2 polarization are two extremes of macrophage activation. M1 macrophages are killing and pro-inflammatory, while M2 macrophages have relatively healing and anti-inflammatory properties [3]. Different polarized macrophages usually show distinct functions in viral infection. For instance, DENV induces high levels of IL-1β and IL-18 from M1 polarized macrophages and causes pyroptosis, whereas M2-polarized macrophages do not produce IL-1β and IL-18 during DENV infection, even with LPS priming [6]. Besides acting as a shelter for JEV infection, macrophages also secrete huge quantities of interferons, chemokines, and pro-inflammatory cytokines, exerting a great impact on the immune response and pathogenesis of JE. Currently, the polarization and immune responses of JEV-infected macrophages at the transcriptomic level have not been characterized.

In this research, we studied the transcriptomic alteration of JEV-infected macrophages by using an in situ infection model. We found that, upon JEV infection, the transcription of 9433 genes significantly changed. Gene Ontology (GO) and Kyoto Encyclopedia of Genes and Genomes (KEGG) analyses revealed that, upon JEV infection, macrophages underwent M1 polarization and showed drastic activation of interferon (IFN), tumor necrosis factor (TNF), interleukin, and chemokine signaling. Moreover, multiform programmed cell death (PCD) pathways, especially apoptosis and pyroptosis, were launched, which was probably caused by extensive DNA damage and oxidative stress. Our work puts forward a new in situ infection model of macrophages and provides a transcriptomic profile of innate immune, inflammatory, and cell death responses to JEV infections in macrophages.

## 2. Materials and Methods

### 2.1. Cells and Virus

C6/36 cells (*Aedes albopictus* lava cells) were grown in an RPMI 1640 medium (Gibco, Grand Island, USA) supplemented with 10% fetal bovine serum (FBS, PAN, Adenbach, Germany) and maintained at 28 °C. Vero cells (African green monkey kidney cells) were raised in a minimum essential medium (MEM, Gibco, USA) supplemented with 5% FBS and maintained at 37 °C. 

JEV Beijing strain-1 (JEV) [7] was propagated in C6/36 cells, and the supernatants were gathered at 3 days post-infection (dpi) and at 4 dpi. The supernatants were allocated and stored at -80 °C for later use. The virus titer was determined by a plaque assay on Vero cells.

### 2.2. Mice and in Situ Infection of Peritoneal Macrophages

Four-week-old female C57BL/6J mice were purchased from Vital River Laboratories (China) and raised in a specific pathogen-free animal facility at Capital Medical University, China. For the in situ infection of peritoneal macrophages, the mice were randomly divided into a mock-treated group (*n* = 5) and a JEV-infected group (*n* = 5). Mice in the mock-treated group were intraperitoneally (i.p.) injected with 200 μL phosphate-buffered saline (PBS) per mouse, while mice in the JEV-infected group were i.p. injected with 10^5^ PFU of JEV in 200 μL PBS per mouse. After i.p. injection, the mice were given an abdominal massage for 2–3 min to allow even contact of the peritoneal macrophages and viruses.

### 2.3. Isolation of Peritoneal Macrophages

At 24 h post infection (hpi), the mice were euthanized, and blood samples were collected for serum isolation, followed by an i.p. injection with 4 mL of PBS per mouse and an abdominal massage for 3 min, before being left to stand for 7 min. Then, the peritoneal lavage was harvested and centrifuged at 2000 rpm for 8 min to collect the cell pellet and supernatant. The cell pellet was washed with PBS. For smear preparation, each cell pellet per mouse was resuspended in 1 mL of PBS. For total RNA extraction, each cell pellet per mouse was homogenized in 1 mL of TransZol (ET101-01, TransGen).

### 2.4. Smear Preparations of Peritoneal Macrophages

Peritoneal macrophages collected from a single mouse were resuspended in 1 mL of PBS; 0.1 mL of cell resuspension was equally smeared on a slide, dried with blowing air to ensure tight adhesion, and then fixed with 4% paraformaldehyde (PFA). After fixation, the slides were washed with PBS to remove residual PFA, followed by blowing air to ensure dryness and tight adhesion. The dried slides were maintained at −20 °C.

### 2.5. Viral Load Detection in Peritoneal Lavages and Sera

The JEV genome RNA in peritoneal lavages and sera was extracted by using a commercial EasyPure Viral DNA/RNA Kit (TransGen, Beijing, China). A previously reported [8] FAM-TAMRA probe-based qRT–PCR method was used to quantify the JEV genome RNA copies in the peritoneal lavages and sera.

### 2.6. Total RNA Extraction and Quality Control

The peritoneal macrophages isolated from a single mouse were homogenized in 1 mL of TransZol, and the total RNA was extracted according to the manufacturer’s instruction and dissolved in RNase free water. The concentration and purity of the RNA were determined by a NanoPhotometer, and the RNA integrity was analyzed by an Agilent 2100 bioanalyzer.

### 2.7. New England Biolabs (NEB) General Library Building

The first strand of cDNA was synthesized in an M-MuLV reverse transcriptase system with fragmented mRNA as a template and a random oligonucleotide as a primer; then, the RNA strands were degraded with RNaseH, and the second strand of cDNA was synthesized with dNTPs as substrates in the DNA polymerase I system. The purified double-stranded cDNA then underwent end repair, tail A, and adapter incorporation. The cDNA of 250–300 bp was screened by AMPure XP beads, amplified by PCR, and purified by AMPure XP beads again. Finally, a library was obtained. The kit used in library building was a NEBNext Ultra RNA Library Prep Kit for Illumina. The library was quantified by a Qubit2.0 Fluorometer and diluted to 1.5 ng/uL. Then, an Agilent 2100 Bioanalyzer was used to detect the insert size of the library. When the insert size met expectations, qRT–PCR was used to accurately quantify the effective concentration of the library (>2 nM).

### 2.8. Clustering and Sequencing

The clustering of the index-coded samples was performed on a cBot Cluster Generation System using TruSeq PE Cluster Kit v3-cBot-HS (Illumina, San Diego, USA) according to the manufacturer’s instructions. After cluster generation, the library preparations were sequenced on an Illumina Novaseq platform, and 150 bp paired-end reads were generated.

### 2.9. Sequencing Data Processing

For quality control, raw data (raw reads) in the fastq format were first processed through in-house Perl scripts. In this step, clean data (clean reads) were obtained by removing reads containing an adapter, reads containing ploy-N, and reads of a low-quality. Q20, Q30, GC content, and the clean data were then calculated. All the downstream analyses were based on the clean data.

For read mapping, the reference genome (GRCm38.p6) and gene model annotation files (GCA_000001635.8) were downloaded from the genome website directly. An index of the reference genome was built using Hisat2 v2.0.5, and paired-end clean reads were aligned to the reference genome using Hisat2 v2.0.5.

For gene expression quantification, featureCounts v1.5.0-p3 was used to count the read numbers mapped to each gene. Then, the FPKM of each gene was calculated based on the length of the gene, and the count of reads mapped to this gene. The FPKM (the expected number of fragments per kilobase of the transcript sequence per million base pairs sequenced) considers the effect of the sequencing depth and gene length for the read count at the same time and is currently the most commonly used method for estimating gene expression levels.

A differential expression analysis of the two groups was performed using the DESeq2 R package (1.16.1). The resulting *p*-values were adjusted using Benjamini and Hochberg’s approach for controlling the false discovery rate. Genes with an adjusted *p*-value <0.05 and log_2_|Fold Change| > 0 were deemed to be differentially expressed [9].

A Gene Ontology (GO) enrichment analysis of differentially expressed genes (DEGs) was implemented by the cluster Profiler R package, in which gene length bias was corrected. GO terms with corrected *p*-values less than 0.05 were considered significantly enriched by the DEGs. KEGG is a database resource for understanding the high-level functions and utilities of the biological system (http://www.genome.jp/kegg/). We used the cluster Profiler R package to test the statistical enrichment of DEGs in the KEGG pathways.

### 2.10. Immunofluorescent (IF) Staining

For immunofluorescent (IF) staining, the smears of macrophages isolated from mock-treated mice and JEV-infected mice were subjected to membrane permeability with 0.5% Triton (in PBS) for 5 min and blockage with 1% bovine serum albumin (BSA) for 1 h at 4 °C and then incubated with specific primary antibodies (1:250–1:300) at 4 °C overnight, followed by incubation with fluorescent secondary antibodies (1:400) at room temperature for 50 min.

### 2.11. TUNEL Staining

For TUNEL staining, smears of the macrophages isolated from the mock-treated mice and JEV-infected mice were subjected to a membrane permeability treatment with 0.5% Triton (in PBS) for 5 min, followed by a PBS wash and TUNEL staining with a One Step TUNEL Apoptosis Assay Kit (C1086, Beyotime, Shanghai, China), according to the manufacturer’s instruction.

### 2.12. Ethical Statement

All the animal experiments were approved by the Experimental Animal Welfare and Animal Ethics Committee of Capital Medical University, China (permission code: AEEI-2019-050; permission date: 9 April 2019 ).

## 3. Results

### 3.1. In Situ JEV Infection Model of the Macrophages and Transcriptomic Research Design.

**Figure 1:**
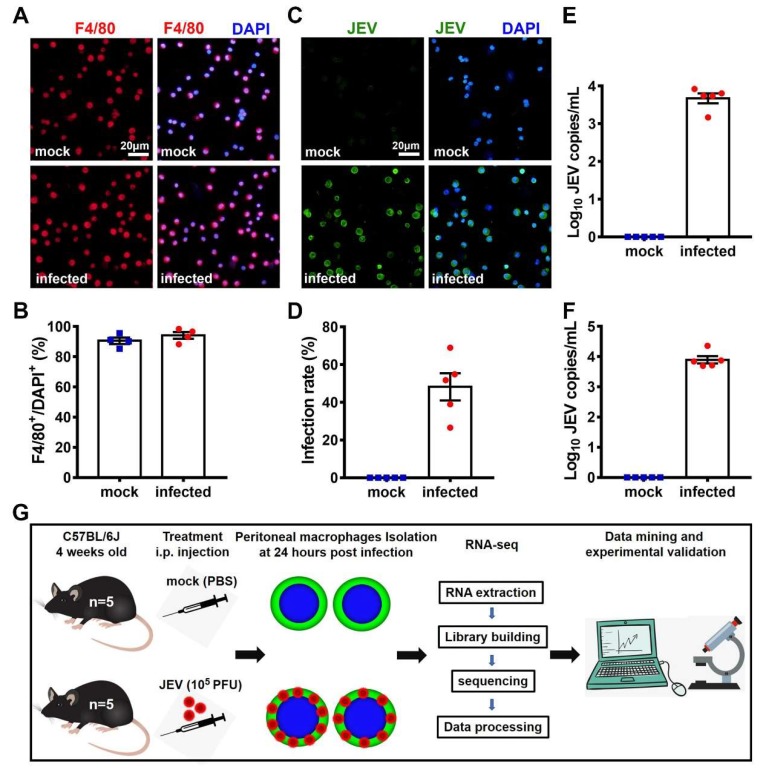
Infection of the peritoneal macrophages and transcriptomic research design. Four-week-old female C57BL/6J mice were randomly divided into a mock-treated group (*n* = 5) and a Japanese encephalitis virus (JEV)-infected group (*n* = 5). At 24 hpi, mice were euthanized, and their sera, peritoneal macrophages, and peritoneal lavages were collected. (**A**) Immunofluorescent (IF) staining of F4/80 (red), a specific marker for macrophages. DAPI denotes the nucleus; scale bar = 20 μm. (**B**) Macrophage purity is shown as the proportion of F4/80^+^ cells. (**C**) IF staining of JEV antigens (green). DAPI denotes the nucleus; scale bar = 20 μm. (**D**) Infection rate of the peritoneal macrophages. (**E**) JEV RNA load in peritoneal lavages (each mouse was washed with 4 mL PBS). (**F**) JEV RNA load in mouse sera. (**G**) Schematic description of the transcriptomic research design. The quantitative results are shown as the mean ± SEM, and each dot denotes an independent mouse.

To establish the in situ JEV infection model of the macrophages, four-week-old female C57BL/6J mice were randomly divided into a mock-treated group and a JEV-infected group (*n* = 5 for each group), in which each mouse was i.p. injected with 200 μL PBS (mock) or 10^5^ PFU of JEV in 200 μL PBS (JEV). At 24 hpi, the mice were euthanized, and their sera, peritoneal macrophages, and peritoneal lavages were extracted. The purity of the isolated peritoneal cells was examined by the IF staining of F4/80, a specific marker of macrophages (Figure 1A). The purity of the peritoneal macrophages isolated from the mock-treated and JEV-infected mice was 91% and 94%, respectively, indicating that most of the peritoneal cells isolated were macrophages (Figure 1B). The infection rate of the JEV-infected macrophages was 50%, which was examined by the IF staining of JEV antigens (Figure 1C,D). The JEV RNA load in the peritoneal lavage and mouse serum was 10^3.7^ copies/mL (Figure 1E) and 10^3.9^ copies/mL (Figure 1F), respectively. These data suggest that the in situ macrophage infection model worked well and could be used for investigating the transcriptomic characteristics of the mock-treated (*n* = 5) and the JEV-infected (*n* = 5) in situ macrophages (Figure 1G).

### 3.2. Quality Control of Sequencing Data 

**Figure 2:**
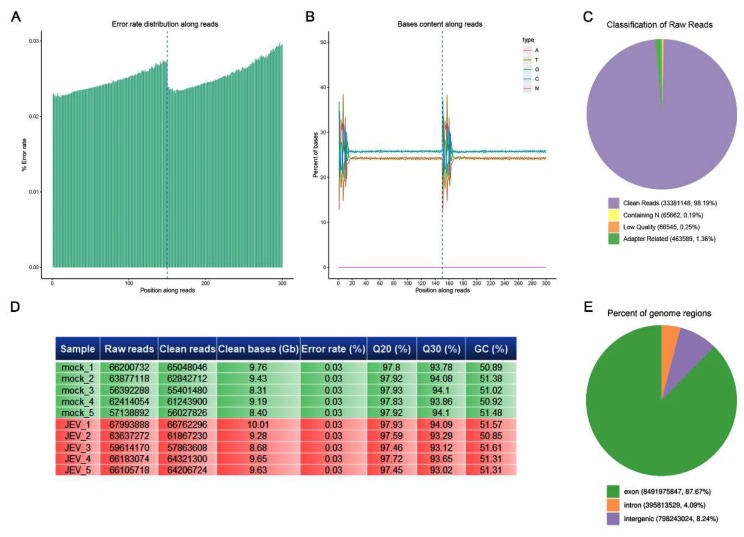
Quality control of sequencing. New England Biolabs (NEB) general libraries were sequenced on an Illumina sequencing platform, and 150 bp paired-end reads were generated. (**A**) Error rate distribution along reads. (**B**) Base content along reads. (**C**) Classification of raw reads. (**D**) Tabular summary of the essential parameters of the sequence data. (**E**) Percentage of the different regions mapped to the genomes.

First, we analyzed the data quality of the RNA-seq. The error rate of sequencing increases with the elongation of the sequenced reads, which is caused by the consumption of chemical reagents in the sequencing process and is a feature of the Illumina high-throughput sequencing platform. The sequencing error rate of all our samples was below 0.03% (Figure 2A). The proportion of guanine (G) and cytosine (C) in the nucleotide sequence is called the GC content. The GC content has a certain specificity among species, but due to the 6-bp random primers used in the reverse transcription process, the first few bases will have a certain preference in nucleotide composition, causing normal fluctuation, and the GC content in the rest of the sequence tends to be stable. For New England Biolabs (NEB) general library building, due to random fragmentation and double chain complementation, the GC and AT contents of each read should theoretically be equal and remain stable as a horizontal line along the whole sequencing process. The GC content of all our samples obeyed this rule (Figure 2B). The original data obtained by sequencing contained a very small number of reads with sequencing adapters of low sequencing quality. To ensure the quality and reliability of the data analysis, it is necessary to filter the original data, including removing the reads with an adapter containing n (n indicates that the base information cannot be determined) or of a low-quality. The proportion of clean reads in all the reads of our samples exceeded 98%, indicating a high quality of sequencing (Figure 2C). Figure 2D shows a summary of the sequencing quality of all the samples. We can see that the Q30 (error rate <1/1000) of all bases was 93%–94%, and Q20 (error rate <1/100) exceeded 97%, indicating that the accuracy of sequencing was very high (Figure 2D). According to the mapping results, the proportion of reads mapped to the exon region, intron region, or intergenic region of the genome can be calculated. The proportion of exon-mapped reads in all our samples exceeded 86% (Figure 2E), indicating high-quality genome mapping.

### 3.3. Transcriptomic Profile of JEV-Infected Macrophages

**Figure 3:**
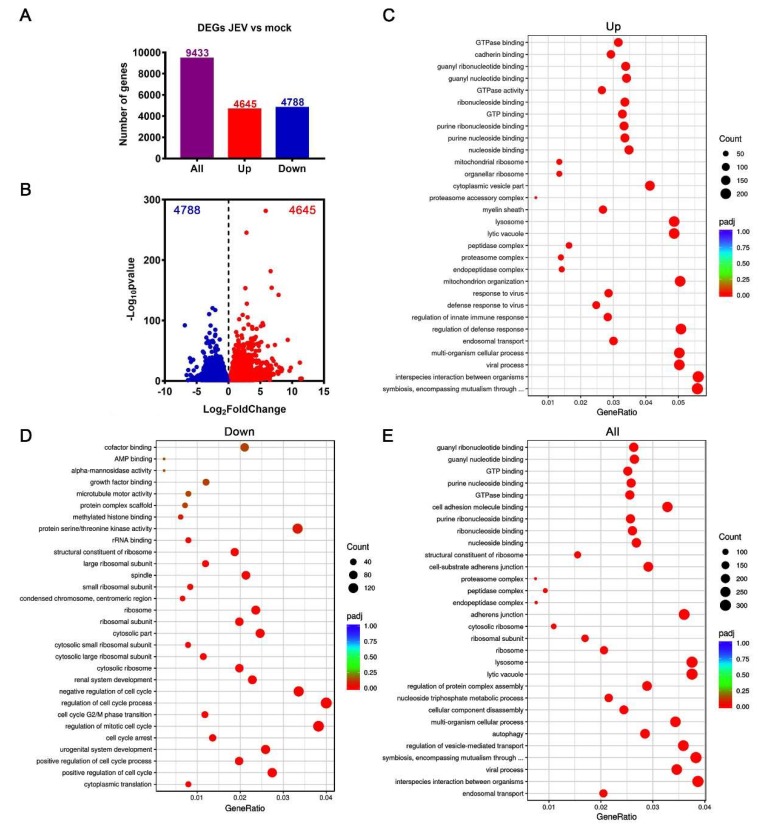
Transcriptomic profile of JEV-infected macrophages. (**A**) Number of significantly changed genes. (**B**) Volcano plot showing significantly upregulated (red dots) and downregulated genes (blue dots). (**C–E**) GO enrichment analysis of upregulated genes (**C**), downregulated genes (**D**), and all changed genes (**E**). The top 30 enriched biological processes were listed based on a ranking of the adjusted *p*-values (padj).

**Figure 4:**
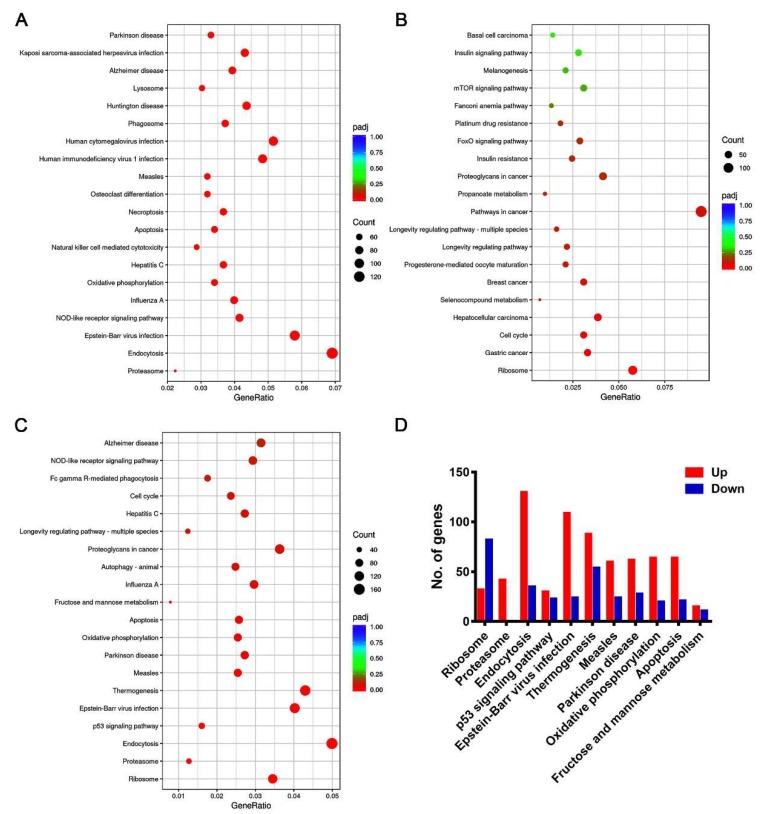
Kyoto Encyclopedia of Genes and Genomes (KEGG) enrichment analysis of JEV-infected macrophages. KEGG enriched pathways of upregulated genes (**A**), downregulated genes (**B**), and all changed genes (**C**); the top 20 pathways were listed based on the ranking of their adjusted *p*-values (padj). (**D**) Bar plot showing the detailed number of up-regulated and down-regulated genes in the top 11 KEGG enriched pathways.

Compared with the mock-treated macrophages, the JEV-infected macrophages showed remarkable transcriptomic alteration. A total of 9433 genes significantly changed, of which, 4645 genes were upregulated and 4788 genes were downregulated (Figure 3A,B). These upregulated genes were mainly involved in the biological processes related to viral infection, innate immune and host defense response to viral infection, symbiotic and interspecies interaction, and endosomal transport by GO analysis (Figure 3C). The downregulated genes were mainly involved in the biological processes related to cell cycle regulation and ribosome organization (Figure 3D). For all changed genes, the GO analysis suggests that they were mainly involved in biological processes such as viral infection, interspecies interactions and symbiosis, vesicle transport, ribosome and lysosomal function, nucleoside triphosphate metabolism, and autophagy (Figure 3E). The KEGG analysis indicated that the upregulated genes were mainly involved in endocytosis, various viral infection, inflammation, apoptosis, and necroptosis pathways (Figure 4A). The downregulated genes were mainly classified as ribosome synthesis genes, cell cycle genes, and various tumor-related pathways (Figure 4B). All changed genes were mainly classified into ribosome and proteasome biosynthesis, endocytosis, p53 signaling pathway (closely related to the DNA damage response and repair and cell cycle regulation), viral infection, apoptosis, and other pathways (Figure 4C,D).

### 3.4. M1 Polarization of JEV-Infected Macrophages

**Figure 5:**
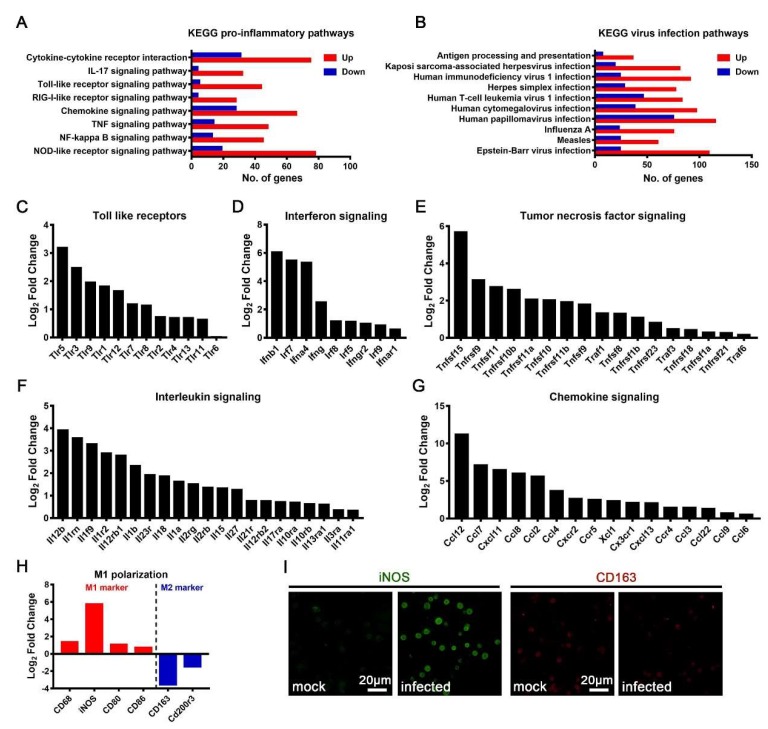
Immune and inflammatory responses in JEV-infected macrophages. A KEGG analysis of the inflammatory related pathways (**A**) and viral infection related pathways (**B**). Significantly up-regulated Toll-like receptor (TLR) genes (**C**), interferon signaling-related genes (**D**), tumor necrosis factor (TNF) signaling-related genes (**E**), interleukin signaling-related genes (**F**), and chemokine and chemokine receptor genes (**G**). (**H**) The transcription level of M1 and M2 polarization-related marker genes. (**I**) The IF staining of inducible nitric oxide synthase (iNOS, marker of M1 polarization, green) and CD163 (marker of M2 polarization, red) in mock-treated and JEV-infected macrophages, scale bar = 20 μm.

Macrophages are innate immune cells that play pivotal roles in the process of anti-infection immune and inflammatory responses. A KEGG analysis of the transcriptomic data showed that after JEV infection, multiple inflammatory responses, including the Nod-like receptor signaling pathway, the NF kappa B signaling pathway, the TNF signaling pathway, the chemokine signaling pathway, the RIG-I like receptor signaling pathway, the Toll-like receptor signaling pathway, the IL-17 signaling pathway, and the cytokine–cytokine receptor interaction pathway, were activated (Figure 5A). Meanwhile, multiple viral infection-related pathways, as well as the antigen processing and presentation pathways, were activated (Figure 5B). Toll-like receptors (TLRs) are important pattern recognition receptors that recognize a variety of pathogen-related molecular patterns and play important roles in the process of anti-infection immune and inflammatory responses. Upon JEV infection, the expression of TLRs was significantly upregulated, especially that of TLR5, TLR3, TLR9, TLR1, TLR12, TLR7, and TLR8 (Figure 5C). Interferon (IFN) is the most important cytokine to mediate the innate antiviral response. Upon JEV infection, the expression of IFNs and the IFN regulatory factors (promoting IFN production) significantly increased (Figure 5D). Remarkably, the expression of type I IFN (IFN-β1 and IFN-α4), type II IFN (IFN-γ), IFNAR1, and IFNGR2 increased significantly (Figure 5D). Tumor necrosis factor (TNF) and interleukin (IL) are two important types of cytokines that mediate inflammation. Upon JEV infection, the expression levels of several TNF superfamily (TNFSF) members and TNF receptor superfamily (TNFRS) members were significantly upregulated, among which TNFSF15 was the most increased (Figure 5E). Upon JEV infection, the expression of IL-12β, IL-1Ra (IL1RN), IL-1f9, IL-1R2, IL-12Rβ1, and IL-1β was significantly increased (Figure 5F). Interestingly, the upregulation of the IL-12 and IL-1 signaling pathway-related genes was the most prominent after JEV infection, suggesting that IL-12 and IL-1 signaling might play important roles in JEV infection (Figure 5F). Chemokines are also important cytokines that attract and activate immune cells to reinforce immune and inflammatory responses. During JEV infection, the expression levels of many chemokines and chemokine receptors increased sharply, among which the most increased were CCL12, CCL7, CXCL11, CCL8, and CCL2 (Figure 5G). Considering the extensive secretion of IFNs, inflammatory cytokines, and chemokines, JEV-infected macrophages might have undergone M1 polarization. To prove this, we analyzed the expression of M1 polarization marker genes in JEV-infected macrophages and found that the expression levels of inducible NO synthase (iNOS), CD68, CD80, and CD86 were significantly upregulated; notably, the expression of iNOS increased by more than 50 times (Figure 5H). Meanwhile, the expression level of M2 polarization marker genes showed significant downregulation, especially CD163, which was downregulated more than 10 times (Figure 5H). In order to verify the M1 polarization of JEV-infected macrophages, immunofluorescence (IF) staining was performed on the isolated macrophages. The expression of iNOS in mock-treated macrophages was very low, but its expression increased significantly after JEV infection (Figure 5I). On the contrary, CD163, representing M2 polarization, decreased after JEV infection (Figure 5I). These results suggest that, upon JEV infection, the macrophages underwent M1 polarization.

### 3.5. JEV Infection Activated Diverse Programmed Cell Death Pathways

**Figure 6:**
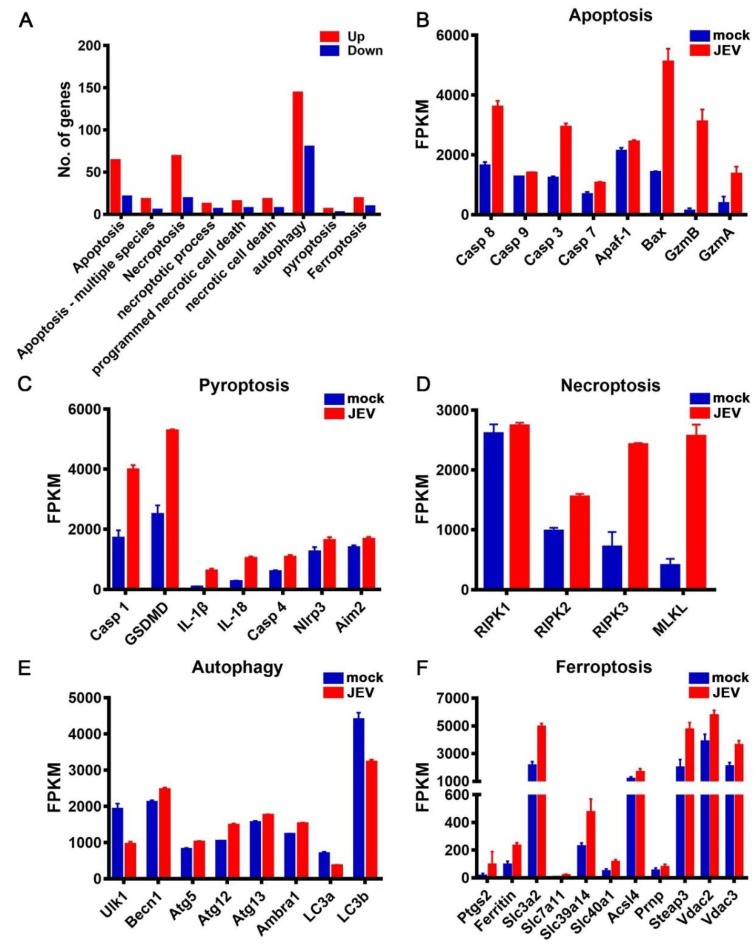
Activation of multiple programmed cell death (PCD) pathways in JEV-infected macrophages. (**A**) The KEGG and GO enrichment analysis of multiple PCD pathways. (**B–F**) Expression level of the crucial genes involved in apoptosis (**B**), pyroptosis (**C**), necroptosis (**D**), autophagy (**E**), and ferroptosis (**F**) in mock-treated (*n* = 5) and JEV-infected (*n* = 5) macrophages; the results are expressed as the mean ± SEM.

Both the GO and KEGG analyses indicated that diverse PCD pathways were conspicuously activated upon JEV infection (Figure 6A). Next, we analyzed the transcription level of the key genes involved in apoptosis, pyroptosis, necroptosis, autophagy, and ferroptosis pathways, respectively. Upon JEV infection, the expression of Caspase-8, Caspase-9, Caspase-3, and Caspase-7 significantly increased, especially Caspase-8 and Caspase-3 (Figure 6B). The expression of several important pro-apoptotic genes also increased significantly after JEV infection, especially Bax, GZMB, and GZMA. The expression of Caspase-1, Gasdermin D (GSDMD), IL-1β, and IL-18, the key genes involved in canonical pyroptosis, significantly increased after JEV infection (Figure 6C). The expression of Caspase-4, the key gene in non-canonical pyroptosis, NLRP3, and Aim2, the key genes that promote pyroptosis, were also found to be significantly upregulated, suggesting that both canonical and non-canonical pathways of pyroptosis might have been activated after JEV infection (Figure 6C). Among the key genes involved in necroptosis, except for RIPK1, the expression levels of RIPK2, RIPK3, and the executive protein MLKL significantly increased, suggesting that the necroptosis pathway might have also been activated after JEV infection (Figure 6D). After JEV infection, the expression of several genes involved in the autophagy pathway was upregulated, but the magnitude of this upregulation was not as large as that of the other PCD pathway genes (Figure 6E). Moreover, the expression of LC3a and LC3b was downregulated after JEV infection (Figure 6E). Therefore, whether autophagy was activated or sequestered after JEV infection needs further experimental verification. Ferroptosis is also a kind of PCD that is iron-dependent. After JEV infection, the expression levels of several genes involved in ferroptosis were significantly upregulated, suggesting that the ferroptosis pathway might also be activated after JEV infection (Figure 6F). 

**Figure 7:**
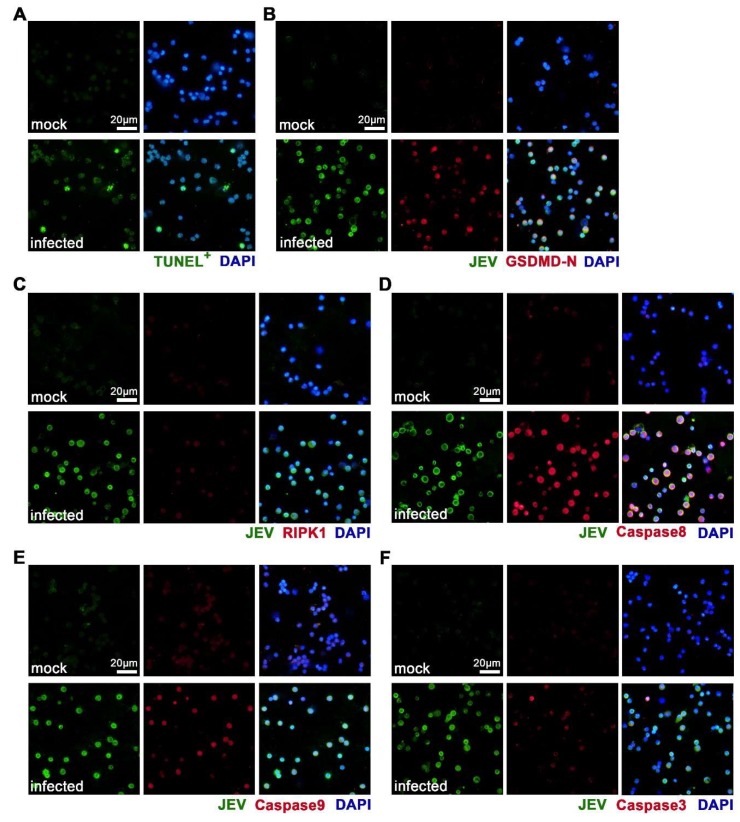
Activation of apoptosis, pyroptosis, and necroptosis in JEV-infected macrophages. Mouse peritoneal macrophages were isolated and made into smears at 24 h post infection. (**A**) TUNEL staining of apoptotic cells. (**B**) IF staining of JEV antigens and Gasdermin D N-terminal (GSDMD-N, pyroptotic marker). (**C**) IF staining of JEV antigens and RIPK1 (necroptotic marker). (**D–F**) Immunofluorescent (IF) staining of JEV antigens and PCD regulating proteins Caspase 8 (**D**), Caspase 9 (**E**), and Caspase 3 (**F**). Scale bar = 20 μm.

We then used IF staining to verify the occurrence of the apoptosis, pyroptosis, and/or necroptosis of JEV-infected in situ macrophages. We found that the mock-treated macrophages showed negative TUNEL staining, indicating that no apoptosis occurred (Figure 7A). As expected, a number of JEV-infected macrophages showed positive TUNEL staining, indicating that apoptosis occurred (Figure 7A). There was no positive staining of GSDMD-N in the mock-treated macrophages, but a large proportion of JEV-infected macrophages showed positive and high expression of GSDMD-N, indicating the occurrence of pyroptosis (Figure 7B). Mock-treated macrophages expressed extremely low levels of RIPK1, while some of the JEV-infected macrophages expressed increased, but still low, levels of RIPK1, suggesting that there might be a low level of necroptosis upon JEV infection (Figure 7C). These results confirm the activated PCD pathways revealed by the transcriptomic analysis and suggest that a large part of the JEV-infected macrophages underwent pyroptosis and apoptosis. We also validated the expression of Caspase8, Caspase9, and Caspase3 by IF staining, which are three crucial proteins that regulate both apoptosis and pyroptosis. In congruence with transcriptomic alteration, all three proteins showed obvious upregulation in JEV-infected macrophages (Figure 7D–F).

### 3.6. JEV Infection Elicited DNA Damage and Repair Dysfunction

**Figure 8:**
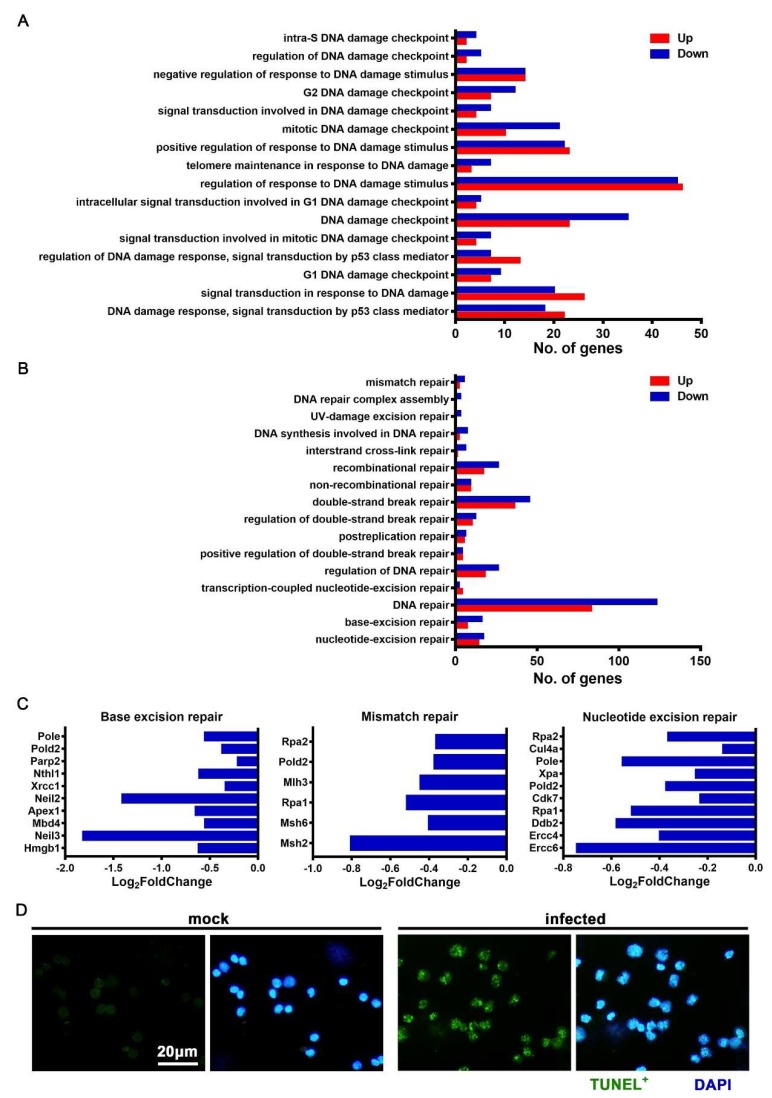
DNA damage and repair dysfunction in JEV-infected macrophages. (**A–B**) Gene Ontology (GO) enrichment analysis of the DNA damage response related pathways (**A**) and DNA damage repair related pathways (**B**). (**C**) Expression of crucial genes involved in base excision repair, mismatch repair, and nucleotide excision repair. (**D**) TUNEL staining of DNA breakages in the mock-treated and JEV-infected macrophages, scale bar = 20 μm.

DNA damage and repair dysfunction are important causes of cell death. We analyzed the DNA damage and repair pathways of JEV-infected macrophages and found that many pathways of DNA damage responses and regulation changed significantly (Figure 8A). In addition, many pathways related to DNA damage repair were inhibited, and the number of downregulated genes was significantly greater than that of the upregulated genes (Figure 8B). Several key genes involved in base excision repair, mismatch repair, and nucleotide excision repair were significantly downregulated after JEV infection (Figure 8C). These results suggest that, after JEV infection, macrophages might have obvious DNA damage, and DNA damage repair capabilities were also significantly sequestered.

In order to verify that the JEV-infected macrophages had obvious DNA damage, we used the TUNEL staining method to mark the end of the broken DNA. We found that the mock-treated macrophages showed no DNA breakage (TUNEL-), while the JEV-infected macrophages showed obvious and extensive DNA breakage (TUNEL+), with multiple dots or small pieces of positive staining in the nucleus (Figure 8D). When DNA damage accumulated to a certain extent and there was obvious positive TUNEL staining of the whole nucleus, cell apoptosis took place (Figure 7A, infected).

### 3.7. JEV Infection Caused Oxidative Stress in Macrophages

**Figure 9:**
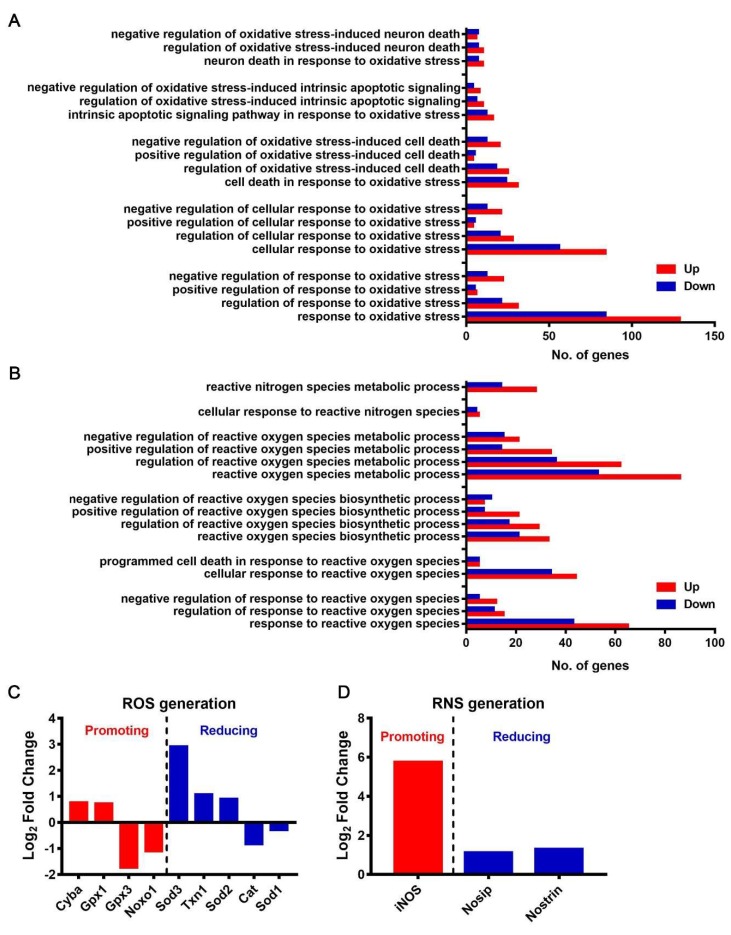
Oxidative stress and reactive oxygen species (ROS)/reactive nitrogen species (RNS) generation in JEV-infected macrophages. (**A**) GO enrichment analysis of multiple pathway responses to oxidative stress. (**B**) GO enrichment analysis of multiple pathways related to the responses to ROS/RNS and the biosynthesis and metabolism of ROS/RNS. (**C**) Expression level of crucial genes promoting (red) or reducing (blue) ROS generation. (**D**) Expression level of crucial genes promoting (red) or reducing (blue) RNS generation.

We found that, upon JEV infection, numerous genes were upregulated in multiple oxidative stress response pathways, suggesting that JEV infection caused oxidative stress in macrophages (Figure 9A). Several oxidative stress-induced cell death or apoptosis pathways were activated (Figure 9A), suggesting that the oxidative stress caused by JEV infection might be an important cause of macrophage death.

Reactive oxygen species (ROS) and reactive nitrogen species (RNS) are important substances that cause oxidative stress in cells. The GO analysis showed that ROS biosynthesis and metabolism-related pathways were activated, and the ROS response pathways, including the PCD pathway, were also activated (Figure 9B). We analyzed the transcription of specific genes that promote or reduce ROS generation. Among the genes that promote ROS generation, Cyba and Gpx1 were upregulated, while Gpx3 and Noxo1 were downregulated. Among the genes that reduce ROS generation, Sod3, Txn1, and Sod2 were upregulated, while Cat and Sod1 were downregulated (Figure 9C). The pathways related to the cellular response to RNS and RNS metabolism showed significant changes, and most genes were upregulated (Figure 9B). We also examined the transcription of specific genes that promote or reduce RNS generation and found that iNOS was the most upregulated gene that promotes RNS generation. Nosip and Nostrin were two upregulated genes that reduced RNS generation (Figure 9D). These results suggest that JEV infection might induce an increased generation of ROS and RNS, resulting in oxidative stress and multiform cell deaths.

## 4. Discussion

JEV is the pathogenic agent of JE, which mainly prevails in Asia and Southeast Asia, threatening the health of two billion people, and, thus, is the most important variety of viral encephalitis in the world. Macrophages are crucial target cells for early JEV infection and play key roles in innate immune and inflammatory responses, as well as trigger adaptive immunity. The transcriptomic dissection of JEV-infected macrophages at early phases of infection will be beneficial to understand early virus–host interaction. Here, we used an in situ macrophage infection model to study the transcriptomic alteration of JEV-infected macrophages and found that JEV-infected macrophages underwent M1 polarization and released a range of immune and inflammatory cytokines. Interestingly, upon JEV infection, almost all the PCD pathways were activated. Further studies revealed extensive DNA damage, increased ROS/RNS generation, and oxidative stress, which might be important causes of the multiform of PCD.

Macrophages usually undergo dynamic molecular and functional transformations after activation. Classical M1 and alternative M2 polarization are the two extremes of macrophage phenotype transformation [10]. The M1 phenotype is pathogen killing and pro-inflammatory and is characterized by the increased production of interferons, pro-inflammatory cytokines such as interleukins (IL-12 and IL-23), tumor necrosis factors, chemokines, and ROS/RNS [11]. In contrast, the M2 phenotype is healing and anti-inflammatory and is characterized by the upregulation of Dectin-1, DC-SIGN, mannose receptor, scavenger receptor A, scavenger receptor B-1, CD163, CCR2, CXCR1, CXCR2, and IL-10 [12]. We discovered that, upon JEV infection, M1 polarization-related genes, including iNOS, CD80, and CD86, were significantly upregulated, while M2 polarization-related genes, including CD163 and CD200R, were downregulated, which was validated by IF staining. Consistent with the M1 phenotype, JEV-infected macrophages showed a significantly elevated expression of TLRs, interferons, pro-inflammatory interleukins, TNF, and chemokines. Interestingly, TLR5, which recognizes bacterial flagellin, was the most increased TLR (9-fold). Though TLR5 is widely reported to be engaged in flagellin recognition, its role in viral infection remains unknown. Considering its remarkable upregulation, TLR5 may play important roles in JEV infection, but this possibility needs to be further investigated. Type I IFNs (IFN-α and IFN-β) were the most increased IFNs. For TNF signaling, the expression of Tnfsf15 (TL1A) was much higher than that of the other genes. Tnfsf15 is mainly expressed by monocytes, macrophages, and dendritic cells in response to stimulation by cytokines, immune complexes, and microorganisms [13]. The receptor of Tnfsf15 is DR3 (Tnfsf25). Besides mediating the activation of NF-κB, Tnfsf15 also promotes the activation of Caspases and apoptosis [14]. Thus, Tnfsf15 may play an important role in early JEV infection. IL-12 and IL-1 were the two most activated interleukin signal pathways, which is consistent with the M1 polarization. CCL12 was the most explosively upregulated chemokine (more than 2000-fold). Although CCL12 (MCP-5) shares the same receptor (CCR2) as CCL2, it may have different biological activity than CCL2 [15,16]. CCL12 is a potent monocyte/macrophage chemoattractant, which also attracts lymphocytes and lung fibrocytes [17,18]. In West Nile virus (WNV) infection, CCL12 is significantly upregulated in the brain, which occurs much earlier than the upregulation of IFN-γ and TNF-α and thus is an early trigger of inflammation in the brain [19]. Currently, the role of CCL12 in JEV infection has not been reported.

Another striking phenomenon during JEV infection was the extensive activation of all currently known PCD pathways, especially apoptosis, pyroptosis, and necroptosis. JEV infection induces apoptosis in various cell types [20,21,22,23,24], while the impact of JEV infection on the apoptosis of macrophages has not yet been reported. Our results show that JEV infection stimulated macrophage apoptosis by activating both extrinsic and intrinsic apoptosis pathways. The apoptosis of JEV-infected macrophages may halt viral replication and reduce inflammatory responses. Besides apoptosis, pyroptosis is another PCD mode of JEV-infected macrophages, which is consistent with previous reports [6]. Unlike apoptosis, pyroptosis is accompanied by the release of IL-1β, IL-18, and other pro-inflammatory cytokines, causing vehement inflammatory responses. Currently, necroptotic death induced by JEV infection has only been reported in neurons [25]. Our results suggest that a number of JEV-infected macrophages underwent necroptosis. Necroptosis is a form of PCD that critically depends on RIPK3 and MLKL and generally manifests with the morphological features of necrosis and is also a pro-inflammatory cell death pattern [26]. The death of JEV-infected macrophages can halt virus propagation and release multiple alarming signals that attract and activate innate and adaptive immune cells, facilitating viral killing and cell debris clearance. However, excessive inflammatory responses may also enforce the pathogenesis of viral infection. How to enhance viral killing and cell debris cleaning and avoid the bystander effects of inflammatory responses is a core problem that needs to be solved.

The extensive activation of PCD might be caused by DNA damage and oxidative stress. The transcriptomic analysis revealed the activation of multiple cell death responses to DNA damage and the sequestration of DNA repair mechanisms. TUNEL staining showed extensive DNA breaks in JEV-infected macrophages. Notably, the unrepaired DNA damage was able to prime the type I IFN system and enhance anti-viral and anti-bacterial responses [27]. Whether the DNA damage observed in the JEV infection is just a passive lesion or an active process that aggrandizes IFN production to enhance antiviral response needs to be further investigated. Oxidative stress is elicited when the balance between ROS generation and clearance is disturbed and is a key player in diverse physiological and pathological processes. It has been reported that JEV infection promotes ROS generation in various cell types [20,28,29,30]. Upon activation, M1 macrophages upregulate iNOS and promote RNS generation. ROS and RNS are both highly reactive oxidative weapons that can effectively kill invading pathogens [31]. However, this killing is non-selective. The overproduction of ROS/RNS can also cause damage to DNA, proteins, lipids, and other biomacromolecules, which usually results in apoptosis, necrosis, and/or other kinds of cell death [31,32,33]. JEV infection causes increased intracellular ROS production and oxidative stress, which induces the apoptosis of human promonocytes and murine neuroblastoma cells [20,29,34]. Consistent with these findings, our transcriptomic analysis revealed that JEV infection stimulated ROS and RNS biosynthesis and caused oxidative stress. Moreover, multiple pathways, including apoptosis and cell death related pathways, were activated. These results suggest that JEV infection induces oxidative stress and that DNA damage may be the important cause of macrophage death.

## 5. Conclusions

JEV infection causes M1 polarization and the activation of multiple PCD pathways. The M1-polarized macrophages showed significantly enhanced innate immune and inflammatory responses to JEV infection. The revitalization of multiform PCD may be caused by extensive DNA damage and oxidative stress induced by JEV infection.
